# RegulonDB version 9.0: high-level integration of gene regulation, coexpression, motif clustering and beyond

**DOI:** 10.1093/nar/gkv1156

**Published:** 2015-11-02

**Authors:** Socorro Gama-Castro, Heladia Salgado, Alberto Santos-Zavaleta, Daniela Ledezma-Tejeida, Luis Muñiz-Rascado, Jair Santiago García-Sotelo, Kevin Alquicira-Hernández, Irma Martínez-Flores, Lucia Pannier, Jaime Abraham Castro-Mondragón, Alejandra Medina-Rivera, Hilda Solano-Lira, César Bonavides-Martínez, Ernesto Pérez-Rueda, Shirley Alquicira-Hernández, Liliana Porrón-Sotelo, Alejandra López-Fuentes, Anastasia Hernández-Koutoucheva, Víctor Del Moral-Chávez, Fabio Rinaldi, Julio Collado-Vides

**Affiliations:** 1 Programa de Genómica Computacional, Centro de Ciencias Genómicas, Universidad Nacional Autónoma de México, A.P. 565-A, Cuernavaca, Morelos 62100, Mexico; 2 UMR_S 1090 TAGC, INSERM, Marseille, 13000 France; 3 Laboratorio Internacional de Investigación sobre el Genoma Humano, Universidad Nacional Autónoma de México, Campus Juriquilla, Boulevard Juriquilla 3001, Juriquilla 76230, Santiago de Querétaro, QRO, Mexico; 4 Departamento de Microbiologia Molecular, IBT, Universidad Nacional Autónoma de México, Cuernavaca, Morelos 62100, Mexico; 5 Institute of Computational Linguistics, University of Zurich, Binzmühlestrasse 14, CH-8050 Zurich, Switzerland

## Abstract

RegulonDB (http://regulondb.ccg.unam.mx) is one of the most useful and important resources on bacterial gene regulation,as it integrates the scattered scientific knowledge of the best-characterized organism, *Escherichia coli* K-12, in a database that organizes large amounts of data. Its electronic format enables researchers to compare their results with the legacy of previous knowledge and supports bioinformatics tools and model building. Here, we summarize our progress with RegulonDB since our last *Nucleic Acids Research* publication describing RegulonDB, in 2013. In addition to maintaining curation up-to-date, we report a collection of 232 interactions with small RNAs affecting 192 genes, and the complete repertoire of 189 Elementary Genetic Sensory-Response units (GENSOR units), integrating the signal, regulatory interactions, and metabolic pathways they govern. These additions represent major progress to a higher level of understanding of regulated processes. We have updated the computationally predicted transcription factors, which total 304 (184 with experimental evidence and 120 from computational predictions); we updated our position-weight matrices and have included tools for clustering them in evolutionary families. We describe our semiautomatic strategy to accelerate curation, including datasets from high-throughput experiments, a novel coexpression distance to search for ‘neighborhood’ genes to known operons and regulons, and computational developments.

## INTRODUCTION

RegulonDB is a relational database that offers, in an organized and computable form, updated knowledge on transcriptional regulation in *Escherichia coli* K-12 (1). RegulonDB, first published in 1998, captures the results of a continuous effort to this day (2). Our curation work also feeds the EcoCyc database (3), which together with RegulonDB are the major sources of organized information for the best-known bacterial genome model organism. For years we have expanded the number of objects and their properties in our database, always enriching the modeling of the molecular components governing transcription initiation, as we strive to keep up-to-date with new methodologies. We have also enriched the modeling of gene regulation, proposing new concepts, such as regulatory phrases (4) and, more recently, GENSOR units (genetic sensory-response units) that link signals, the associated regulatory interactions and the regulated response as metabolic and cellular capabilities (5). Briefly, RegulonDB facilitates access to organized information on the mechanisms of transcription initiation; more precisely, RegulonDB organizes the available information on the shadows and fingerprints of these mechanisms in the genome.

Here we present progress since the last *Nucleic Acids Research* (NAR) paper, published in 2013 (1). We have kept our curation up-to-date, including regulation by small RNAs (sRNAs); we report a rather complete repertoire of elementary GENSOR units, each one integrating the network of regulatory interactions and metabolic pathways affected by one transcription factor (TF). This additional information represents major progress for our focus on integrative approaches to facilitate not only information but also summarized knowledge given the high granularity of most biological processes. We have updated the set of computationally predicted TFs, as well as the high-quality position-weight matrices (PWMs) for each TF with sufficient binding sites, and we have included a novel browser based on a clustering of such matrices that reflects their grouping into TF evolutionary families.

We are well aware that a critical barrier in genomics is how to accelerate access to and processing of the large amounts of information and knowledge that are continuously generated. Curation is a bottleneck for facilitating the capability to digest the tsunami of genomic knowledge. This motivated us to initiate the implementation of assisted curation by means of natural language processing (NLP) strategies, thanks to a collaboration with Dr Fabio Rinaldi, an expert in the field. Our initial results capturing growth conditions are promising, although there is a long way to go. Curation of high-throughput (HT) datasets (i.e., chromatin immunoprecipitation [ChIP] variants, microarrays, gSELEX and transcriptional start site [TSS] mapping) is a delicate issue, since we do not want to dilute the high-quality classical experiments with the massive but more fragmented knowledge that these methodologies produce. Our current solution, as discussed in detail below, is at the crossroads of two paradigms: the classic one of a relatively well-organized genome with promoters and binding sites involved in regulation of transcription initiation, and one inundated by scattered promoters and binding sites, many of which we do not yet know if they are involved in transcriptional regulation.

Another avenue linking RegulonDB data at this time with HT-generated profiles of expression is the capability we have implemented for evaluating the similarity of coexpression of any two genes, based on the COLOMBOS microarray library of experiments. We offer those similarity values for all operons and regulons. Finally, additional computational developments are summarized.

## RESULTS

The RegulonDB version 9.0 release contains all the data described below, the sRNAs, elementary GENSOR units and HT datasets. Literature curation is typically up-to-date within 2 months on average for each release.

## AN UPDATED COLLECTION OF INFORMATION ON REGULATORY sRNAs

Classic regulation of transcription initiation governed by TFs affecting promoter activity has been the major focus of RegulonDB. However, as years have passed we have expanded our curation to include regulation by small metabolites and proteins targeting RNA polymerase directly, as well as regulation by sRNAs. The regulatory potential of sRNAs is magnified when we take into account the fact that some sRNAs regulate the expression of genes themselves involved in regulation of many genes, such as sigma factors (like sigma32), global TFs (like H-NS) and other local TFs (like OmpR) which indirectly affect the expression of numerous genes.

We present an updated, integrative view of the known *E. coli* sRNAs. In our manual curation, we considered only data supported by experimental evidence, with the large majority supported by strong evidence (i.e., based on RT-PCR), except for 10 sRNAs supported by microarray experimental data. A total of 120 sRNAs with 231 total interactions are included in this collection, which all together regulate 192 genes. This collection includes detailed and high-quality information about the known regulatory interactions of sRNAs, such as the binding motifs in the targets.

## COMPREHENSIVE SEMIAUTOMATIC CURATED ELEMENTARY GENSOR UNITS

A GENSOR unit, a short term for ‘genetic sensory-response unit,’ initially defined by Gama Castro *et* *al*. in 2011 (5), is a novel concept that from our perspective places regulatory mechanisms in their natural biological context, as part of a flux of information that starts with a change (appearance of a signal) that elicits a regulated response.

Since the 2013 article, we have updated 45 already-curated GENSOR units and added 144 new GENSOR units, to a total of 189. We defined the boundaries of the GENSOR unit concept and its constituents: currently all GENSOR units are elementary, since they are limited to a single TF, starting with the signal, all reactions from the signal to the effector binding the TF, the effect of the TF active conformation on the regulated genes, the regulated transcription units (TUs), their mRNAs, products and the reactions of these products. If any enzyme is part of a multimeric complex, all the monomers of the complex are added (even if they are not directly regulated by the TF). These 144 new GENSOR units have been curated by a semiautomatic method that starts with a pipeline of programs that extract all information pertinent to a GENSOR unit from the RegulonDB and EcoCyc databases; such data are subsequently manually revised and curated and used to generate the visual map available in RegulonDB. The full methodology, motivation and relevance of this integrative new concept will be published elsewhere (Ledezma *et* *al*., manuscript in preparation).

GENSOR unit components and their interactions place a TF and its regulatory mechanisms in a larger context, providing evidence in many cases for the TF's role in decision-making processes and information flux from the signal to the elicited genetically encoded response.

For the process of GENSOR unit construction, we considered the need to reflect relationships between metabolites where two or more are in the same metabolic pathway only a few reactions apart, specifically in coregulated pathways, since some reactions are not necessarily regulated by the TF that defines a particular GENSOR unit. In RegulonDB version 7.0, we included ‘super-reactions’ to include these reaction gaps. In this most recent version of RegulonDB, we have limited the number of reaction gaps to a maximum of three. Reactions have to be successive and present among EcoCyc's metabolic pathways. Three is the average number of total reactions in EcoCyc pathways; since the median number of reactions is 2, this limit allows more than 50% of the pathways to be completed by reaction gaps in their respective GENSOR unit.

RegulonDB 9.0 hosts a GENSOR unit for each local TF (6) for which there is experimental evidence in the database. A total of 103 TFs have a known effector in RegulonDB, including 25 two-component systems. When available, the four components of the GENSOR unit are highlighted: the signal, the signal processing, the genetic switch and the response. By default, effectors are considered signals, unless a reaction that produces the effector is present in the GENSOR unit, in which case the substrate of that reaction is deemed the signal and the reaction itself becomes part of the signal processing. Directionality of reactions is considered in the identification of the four components, as well as for the addition of reaction gaps.

A total of 78 GENSOR units have their four components highlighted; 119 include the genetic switch and the response, and 2 contain only the genetic switch. We believe this gradient of knowledge is a reflection of both the information that we have yet to discover and the cooperation among TFs to orchestrate complete biological processes. GENSOR units, apart from revealing the precise role of the activity of a TF in cellular metabolism, are the building blocks of larger GENSOR units that will describe decisions encoded in the genome in response to changes in the environment.

**Figure 1.**
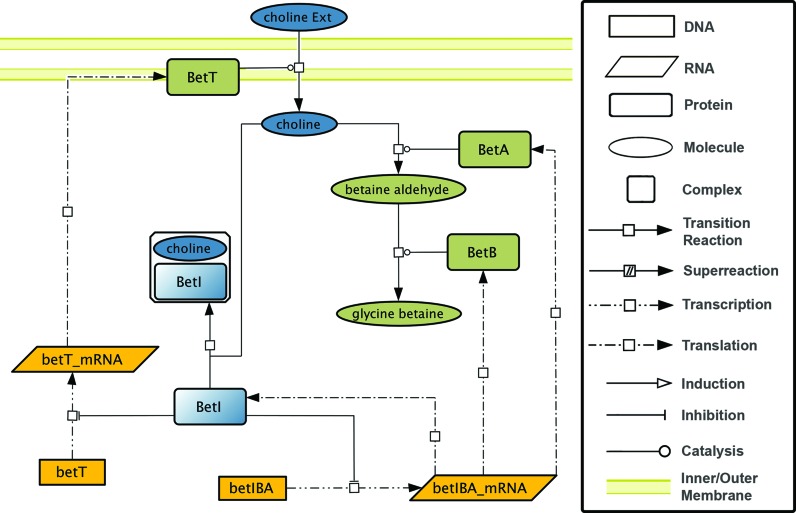
The BetI GENSOR unit. The signal and signal processing, in this case transport of choline through the membrane, are shown in blue. The genetic switch, i.e., repression of *betT* and *betIBA* transcription units, is shown in yellow. The response is shown in green: production of BetT, a choline transporter, and BetA and BetB, enzymes responsible for the utilization of choline.

GENSOR units for which there is sufficient information about their four components have a short written summary describing the higher-level flow of information portrayed. For example, the BetI GENSOR unit (Figure 1) shows that external choline is transported inside the cell, where it binds to BetI and allows the activation of genes involved in the conversion of imported choline to glycine betaine. This information comes from the interactions between the elements in the GENSOR unit, rather than from the elements alone, thus describing the unit with the higher granularity of description, which is more appropriate to understanding physiological and biochemical processes.

The RegulonDB portal has a Web page with the complete list of GENSOR units grouped either by the transduction mechanism or by the signal that initiates a flux of regulated processes (available in the menu under ‘Integrated Views & Tools/RegulonDB Overviews/GENSOR Unit Groups’). Images comply with the Cell Designer (7) graphical notation (8). Cell Designer 4.4 XML format files are available for download for all GENSOR units and their components. Users interested in importing GENSOR units into SBGN-compatible tools can download pure SBML level 2 version 4 XML format files.

We redesigned the Web page for GENSOR units, and this page now contains three sections: the graphical map of the elementary GENSOR unit, its general properties, including the written summary and a section for the properties of each reaction.

## UPDATED TF FAMILIES, POSITION-WEIGHT MATRICES AND THEIR GROUPING IN CLUSTERS

A core component of the transcription machinery is the TF-binding site (TFBS) interaction. In this section, we describe, first our incorporation of an updated set of computationally predicted TFs. Second, we have updated the construction of PWMs for TFs with sufficient known TFBSs. Third, we offer the clustering of TFs based on the similarities of their matrices.

### Updated set of predicted TFs

We updated the set of computationally predicted TFs, based on recent work by Perez-Rueda *et* *al*. (9). A total of 184 TFs experimentally characterized and for which information was deposited in RegulonDB (1) were used as seeds in BLASTP searches against the complete proteome of *E. coli*. *E*-values of ≤1e-6 and a coverage of 70% were required for a TF to be considered a putative TF. In addition, TFs specifically associated with *E. coli* K*-*12 and deposited in the DBD, HAMAP (10), Superfamily DB (11) and PFAM (12) databases were retrieved. Superfamily and family assignations were based on Superfamily annotations (11), PFAM (12) and the Conserved Domain Database (CDD) (11–14). Forty-two groups of paralogs defined by BLASTP (14) comparisons for which the *E*-values were ≤1e-6 and for which coverage was at least 50% of any of the proteins in the alignment were identified in the total set of TFs.

In total, the repertoire of TFs comprises 304 proteins. Of these, 184 are experimentally described in RegulonDB and 120 are predictions. These proteins can be classified in 78 different evolutionary families based on PFAM, CDD and Superfamily annotations. The most abundant family (LysR) entails 46 proteins (15% of the total number of TFs), although for almost 50% of these there is not any experimental evidence. Two additional large families were also identified, AraC/XylS (26 proteins) and GntR (20 proteins). An important improvement of the previous predictions is the elimination of false positives, such as for transposases and integrases.

Of the 184 experimentally described TFs, those for which we have identified binding sites are the subject for PWM construction and clustering, as described in the following section.

### Updated set of PWMs

A minimum of four annotated TFBSs is required for building a motif in the form of a PWM. There were enough sites to build a motif for 93 TFs, 7 more than in the previous version; the full set of sites include 3195 TF → gene regulatory interactions. Using different sequence lengths (variation of ±4 bp around the annotated binding site length), programs (MEME and consensus) and background models (orders 0 and 1), we evaluated the different motifs available for each TF and selected the one with the best quality (15). At the time of the RegulonDB version 8.0 release, we had evaluated the quality of PWMs by taking into account (i) the information content conservation across the PWM; (ii) the false-positive rate for recovering 70% of the annotated sites; (iii) the difference between the observed distribution of scores in the upstream regions on *E. coli* K-12 versus the theoretical distribution; and (iv) the level of overfitting of the PWM to the original sequences used to build it (1).

We were able to obtain a high-quality matrix for 60% of the TFs, 10% more than in the previous version. This version includes motifs for seven TFs that fulfilled only the requirement for four binding sites, plus nine TFs for which the quality increased from low to good. A flat file with the PWMs in consensus format was added in the downloads page of the website. Additionally, in the ‘Integrated Views & Tools’ section, a browser allows navigation through each TF and the distributions that support the quality of the PWMs.

Using the evaluated set of motifs, we found 16 207 predicted binding sites in the upstream region of the *E. coli* K-12 MG1655 *uid57779* genes, where upstream regions are defined as −400 bp upstream to 50 bp downstream from the start codon (16,17). This set of predicted binding sites corresponds to 12 574 TF → gene regulatory interactions; this represents a recovery of 52% of the 1592 annotated regulatory interactions in the database for the 93 TFs for which we have a PWM, which represents a 9% improvement from the previous RegulonDB version. If only TFs with a good-quality PWM are taken into account, the total number of predicted TF → gene interactions is 8714, recovering 672 (57%) of annotated interactions for this TF subset. The TFBS predictions can be obtained from the ‘Dataset’ menu in the ‘Computational Prediction’ section.

### Clustering of PWMs

TFs belong to evolutionarily related families, where members of the same family tend to share a significant similarity of protein domains that bind to DNA, which in bacteria are most frequently helix-turn-helix motifs.

The 93 PWMs available in RegulonDB, built as mentioned before, were analyzed with the program *matrix-clustering* (16), a tool that groups similar PWMs. Given the high similarity of motifs of proteins of the same family, this program can be used to identify TF-binding motifs (TFBMs) that belong to phylogenetically related TFs or DNA-binding proteins that recognize similar DNA sequences. The clustering can be displayed as a collection of hierarchical trees (forest), where each tree represents a cluster with its global alignment of PWMs. Additionally, a heat map representation with an all-versus-all PWMs comparison is also now possible.

We found 47 clusters formed by PWMs corresponding exclusively to TFs of the same family (e.g. AraC, LacI, NarL, GntR and NagC). The alignments of these PWMs show the conserved and non-conserved positions between them. These groups of PWMs are summarized as Familial Binding Profiles (FBP) (14), a general PWM that represents a collection of similar motifs highlighting the similar positions of the clustered PWMs, which allow us to potentially have one FBP for each TF family.

The PWMs were grouped as follows: (i) all the motifs were compared to each other using two metrics to measure their similarity (the Pearson correlation coefficient and a normalized version of the Pearson correlation relative to the width of the match between two aligned PWMs) (18,19). (ii) The motifs were grouped with hierarchical clustering, using the standard UPGMA method (http://arxiv.org/abs/1105.0121). (iii) The hierarchical tree was cut using as the threshold a combination of different metrics values; the tree is cut in a collection of trees (a forest). (iv) Each tree is used as a guide to create a progressive alignment of the PWMs. (v) The clusters are represented both as trees and as heat maps (see http://www.rsat.eu/).

**Figure 2.**
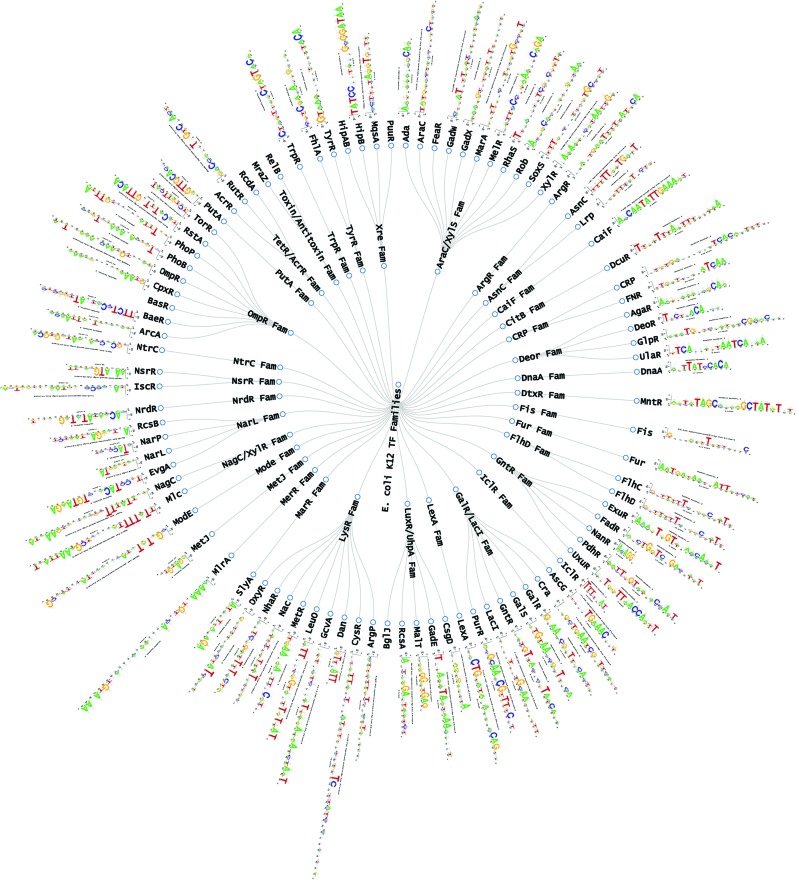
Circular Browser. Transcription factors (TF) classified in their evolutionary families, based on PFAM, CDD and Superfamily annotations.

The browser that enables the user to see the collection of PWMs in a hierarchical tree is available via the ‘Integrated Views & Tools’ menu, in the ‘Browse RegulonDB’ section in the ‘Clustering of RegulonDB PWMs’ option. Also in the same section, there is a link to display a circular browser (Figure 2), developed with the D3.js JavaScript library (http://d3js.org/), that integrates the information from families, the TFs and their PWMs.

## IMPLEMENTING A SEMIASSISTED CURATION STRATEGY

Given the large amount of biological data generated day by day as a result of research in various laboratories, manual curation represents a bottleneck to facilitate access to knowledge in an organized way. We therefore have initiated the implementation of NLP methods in collaboration with the OntoGene group, to enhance the efficiency of curation to keep up with the flood of knowledge and publications, as reported recently (20).

We developed an *ad hoc* interface called ODIN (The OntoGene Document INspector) to curate the literature supporting the knowledge in RegulonDB. The input for ODIN entails full papers, and the output is an interface with several tools to facilitate their curation. We have initiated the process of assisted, or semiautomatic, curation in a very cautious manner, focusing on missing pieces of knowledge, such as growth conditions (GCs) under which specific regulatory interactions (RIs) have been identified.

To do so, filters were created that display in ODIN only those sentences in a paper that contain the data we need to curate; therefore, we do not have to read the full article, but only the phrases that should contain the RIs and GCs. The data we have curated in the traditional way, as is the case with RIs, serve as a control to benchmark this new method. In the case of OxyR, we identified all 20 RIs (100%) that had been previously curated, and we identified the GCs for 16 of them (20).

After reporting the work of OxyR, we used the same strategy to identify the GCs of SoxR and SoxS RIs; we identified 27 of the 28 (96%) RIs of SoxS and obtained the GCs for 13 of them. This lower number may be due to non-specified growth conditions reported in the papers, such as when performing *in vitro* experiments for overexpression of TFs. We also identified 3 out of 3 RIs of SoxR and the GC for 2 of them (see Table S1 in supplementary material). Therefore, now we have in RegulonDB the GCs for 31 RIs, including the OxyR GC-RI pairs, and we will continue to work with other TFs to identify their GC-RI pairs. We will use these results for a cyclic improvement of our assisted curation strategies.

This semiautomatic process enables us to increase the efficiency of curation; however, currently there is a total of 3195 RIs for 199 TFs. This shows the long way we have to go in order to curate all GCs for the RIs. These numbers make clear the necessity for implementation of an assisted curation strategy. We are also motivated to implement NLP filters, not only for new properties but also for a more precise and comprehensive curation, as in the case for methods associated with evidence codes.

### Identification and annotation of methods

All data added to the database contain the evidence that demonstrates the existence of each object or interaction of regulation. We use a set of evidence codes (see http://regulondb.ccg.unam.mx/evidenceclasification) for this purpose. These evidence codes are derived from more than one method that is reported in the literature, as in the case of the evidence to identify TSSs of promoters, ‘Transcription initiation mapping,’ that could be related to the methods of primer extension, S1 mapping or 5’-RACE, among others. This level of description is common to major resources, such as the GO (Gene Ontology) and EcoCyc databases.

A virtue within RegulonDB that we implemented since 2008 is, on the one hand, a simplified classification of ‘weak’ and ‘strong’ levels of confidence for all evidence sources. Strong confidence essentially requires physical evidence for the existence of the object or interaction (21). What is most interesting is the specific algebra of the combinations of evidence that can be considered independent from each other and therefore can be added to increase the overall degree of confidence for an object or interaction, including cross-validation of strong confidence supporting the ‘confirmed’ level of confidence (22).

Motivated by this previous work, we started a project with the group OntoGene to extract the methods for all objects and interactions contained in RegulonDB. We initiated the project by identifying the experimental methods of primer extension and northern blotting. These methods are the most easy to identify by text-mining methods, because very specific words are used to describe them, as opposed to other methods. Northern blotting is commonly used to identify TUs, whereas primer extension is used to identify TSSs of promoters. The objects that were identified with these methods are listed in RegulonDB with strong evidence codes. We took all the papers that are linked to only one promoter or one TU in RegulonDB, where the promoter or TU had the evidence code for ‘Transcription initiation mapping’ and ‘Length of transcript experimentally determined,’ respectively. Subsequently, using ODIN filters, we did a search for the words ‘primer extension’ and ‘northern blot’ in each set of papers. To increase the confidence in these text-mining strategies, we included the requirement that in the phrase(s) that identifies the method, the name of the TU/promoter has to be present too. Of course, we read all these phrases and checked if they were correct. We identified the method of primer extension for 227 promoters and that of northern blotting for 110 TUs. We plan to expand these strategies to extract additional methods and other objects and knowledge from the literature.

## CURATION OF HIGH-THROUGHPUT DATASETS

HT experiments generate a large number of scattered fragments of knowledge. By default, we add such information, with peaks already processed by the authors of the curated papers, as datasets separated from the database, but available, for instance, for display as tracks as part of the different resources in RegulonDB. Our manual curation efforts are focused on extracting the subset of objects (binding sites and promoters) that have additional evidence supporting them, as well as to combine different experiments that congruently support an object. For instance, ChIP-based experiments identify sites that occur within coding regions (23), which may have nothing to do with transcriptional regulation or at least there is not yet evidence of such involvement. Similar concerns may be raised with TSSs identification.

In some cases, a subset of the results is subject to further analysis, such as EMSA, footprinting, northern blotting and/or matrix analyses for site identification. Currently, we add a regulatory interaction in the database only for sites with strong evidence for TFBSs-validated sites (22) and where additional knowledge assigns the function of the TF on the regulated gene, such as ChIP-exo complemented with RNA-seq analysis (24,25). This illustrates the rationale of our approach to combine the data of different HT experiments to integrate these new data with existing knowledge in the database.

There are more than 38 transcriptional regulators (TFs) whose sites have been identified by ChIP methodologies, and this number may increase to 200 TFs for which genomic SELEX screening has been done (26), with data available and published for 17 TFs. A summary of the currently curated datasets is shown in Supplementary Tables S2 and S3. Extraction and curation of this type of data is particularly difficult, because the generated information shows a great variety in terms of formats of the results, and only the central peaks around the binding site for a TF are shown (e.g. 200–300 nt).

There are several HT-dedicated repositories and resources of microbial experimental results, such as TBDB (27), CollectTF (28) and RegTransBase (29), which hold curated motif data from HTs sources with links to GEO from NCBI (30) and to ArrayExpress from EMBL-EBI (31); databases with expression profiles, such as COLOMBOS (32), which offers a variety of tools for analysis, M3D (33) and GenExpDB (http://genexpdb.ou.edu/main/). For a broader context of these resources and many more related to gene regulation, users can visit our link to additional resources (http://regulondb.ccg.unam.mx/menu/about_regulondb/additional_resources/index.jsp).

Our work does not duplicate those efforts, since our main goal is to detect evidence that can be added to either existing objects in RegulonDB and/or that can be combined to support knowledge of higher granularity.

### HT data generated by gSELEX and ChIP-exo

We curated in RegulonDB ChIP-chip data for the PurR regulon by using validation data (22). For gSELEX, we generated two tables, one with raw data (data gSELEX peaks) and the other for cross-data comparisons, i.e., gSELEX and microarrays data for H-NS and LeuO (34); gSELEX and consensus sequences for the transcription factor CRP (35). Only a few cases were uploaded to the database from ChIP-exo plus RNA-seq analysis (24,25). The results generated for both methodologies are summarized in Supplementary Tables S2 and S3.

### HT dataset for TSSs under three conditions

The dataset for 14 868 TSSs from the Storz lab has been curated. It includes 5495 TSSs corresponding to potential antisense RNAs (asRNAs). These data were generated from RNA-seq and prediction algorithms under three different biological conditions: the MG1655 wild-type strain grown to exponential phase or stationary phase in LB medium as well as the wild-type strain grown to exponential phase in M63 minimal glucose medium (36) (see also http://regulondb.ccg.unam.mx/menu/download/high_throughput_datasets/index.jsp).

## COEXPRESSION DISTANCE AROUND THE REGULATORY NETWORK

One of the extensive uses of HT technologies is for the development of global expression profiles. As mentioned before, dedicated databases with information on *E. coli* include COLOMBOS (32), M3D (33) and GenExpDB (http://genexpdb.ou.edu/main/).

For years, RegulonDB has offered links that allow users to upload gene sets to search for their expression profiles in COLOMBOS (www.colombos.net). In addition to these links, we have implemented tools for a full comparison of expression of groups of genes across all conditions.

The ‘Coexpression’ page can be reached directly from the search option. A single query gene or a group of genes are added either manually, based on the set of interest to the user, or are automatically uploaded as a collection of genes defining operons or regulons (from their corresponding pages). The result will be a list of the top 20 genes (the default quantity) that have the highest similarity in coexpression, from the set of all experiments present in COLOMBOS across all conditions. There is a single best list for each one of the genes in the input list, which can be browsed on the ‘Coexpression’ page. These lists include relevant information for the input genes, i.e. the gene product name, the operon to which the gene belongs, the regulators for which the gene has binding sites, and ontological classes of processes in which the gene participates. In the next release of RegulonDB, in an additional section, we will show coexpression by providing color charts to facilitate visualization.

In addition, in the most recent RegulonDB release, we offer a coexpression overview for two groups of input genes: operons and regulons. For dual regulators, regulons are also separated into what we call ‘strict regulons,’ that is to say, groups of target genes subject to the same effect (activator, repressor or dual effect) by a TF. For each group, i.e. each operon, regulon or strict regulon, we display a browser containing the following sections: the name of the group, the genes contained in the group, a ‘coexpression matrix,’ the ‘coexpression distribution’ of the group, and the ‘top best coexpressed’ genes. The ‘coexpression matrix’ section enables the user to see the coexpression values of genes with other genes within the group, and the ‘coexpression distribution’ section shows a plot of the probability density distribution of the coexpression values of the genes within the group, contrasted with a background. For example, the coexpression distribution of the strict regulon ‘CRP,+’ shows the coexpression probability density distribution of all genes activated by CRP with each other, in contrast with the coexpression probability density distribution of all remaining genes in the genome. The ‘top best coexpressed’ section offers a list of additional genes that show the highest coexpression with the genes in the group.

Additional genes that are most highly coexpressed with a group of genes are identified by calculating the top best-scoring medians of the set of coexpression values of any additional gene with each gene of the group. This is certainly an interesting question for any set of query genes, but it is computationally intensive, since for every pair of input genes, we need to identify the intersection of output coexpressed genes. We have therefore precalculated the group values for operons and regulons.

To quantify coexpression for all combinations of gene pairs, we implemented a rank-based approach, using data available in COLOMBOS version 2.0, which contains expression profiles of 2470 different, contrasting conditions. The method and results will be described in detail in a paper to be submitted by Pannier *et* *al*. Gene coexpression is typically quantified by pairwise correlation analysis across large expression compendia. However, these analyses are difficult to interpret because of the highly variable distributions of such correlation coefficients. We use a rank-based approach that normalizes the differences between the range of correlation coefficients between genes, which allows comparisons of coexpression strengths among genes despite the large variability of expression values.

## COMPUTATIONAL ADDITIONS

In order to facilitate searching for information, we implemented a free word-searching tool based in Elastic Search (https://www.elastic.co). This tool enables identification of synonyms for any object, in order by relevance and highlights the searched elements.

### New features of our website

#### Search results

A new view was added to the display of search results by regulon, at the request of our users. When the user selects ‘regulon search’ without giving a term, all the regulons are displayed in a table with the regulon name, the total regulated genes, the total regulated operons, the total binding sites and the total regulatory interactions. The user can sort using any column of this table.

#### Gene page

We created a new section named ‘Elements in the selected gene context region unrelated to any TU in RegulonDB.’ In this section, users can find biological objects in the vicinity of a gene that are not part of its TU, such as the many TSSs near the *micF* gene, to mention one example. In addition, the same gene page in the section called ‘Operon arrangement’ has links to the operon page. Each promoter is linked with the corresponding TU that it transcribes.

#### Sigmulon page

We have included the sigma signal transduction map with a link showing the details of the reactions contained in the map.

#### Datasets

In the submenu related to the datasets, included in downloads, we have integrated new information related to the TSSs experimentally determined in the laboratory of Dr Morett. The TSSs are included in the file named ‘High-throughput transcription initiation mapping. Illumina directional RNA-seq experiments where total RNA received different treatments to enrich for 5′-monophosphate or 5′-triphosphate ends.’ These objects are included in the new section ‘Elements in the selected gene context region unrelated to any TU in RegulonDB,’ previously described.

#### Impact of RegulonDB

**Figure 3.**
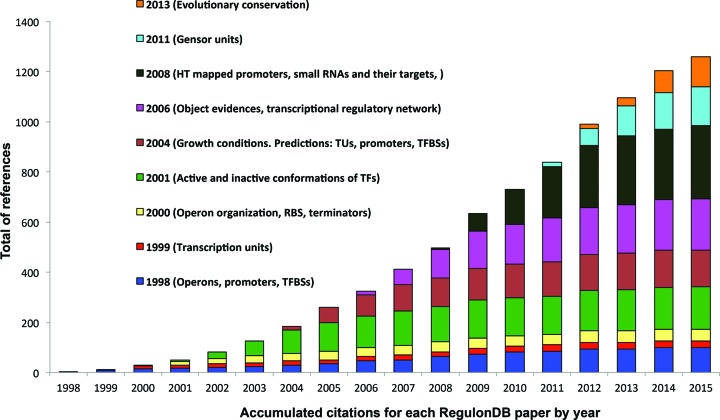
Impact of RegulonDB. Accumulated citations for each RegulonDB paper by year and the concomitant expansion of domains of the biology that we curate.

Figure 3 shows the accumulated citations for each RegulonDB paper by year and the concomitant expansion of new objects and properties related to the regulation of gene expression that we curate. RegulonDB plays a central role in the development and testing of novel approaches of gene regulation in bioinformatics, comparative genomics, and systems biology, and it is the model to inspire similar approaches and studies for any other organism, including pathogenic bacteria (37–39). Evidence of its usefulness is apparent from the more than 1200 citations in published articles, in addition to the many citations for the EcoCyc database, which incorporates our curation work. Within the ‘Features’ menu, we have added this type of information, showing the impact of RegulonDB, such as the number and type of journals for publications that have cited our RegulonDB-related publications.

#### Releases

The release that corresponds to this paper is version 9.0. Major changes to the overall navigation and structure of the main pages have been made, offering more structured access to the data, based on the two dominant types of users: biologists, usually conducting individual search queries, and those interested in data collections.

## CONCLUSIONS

**Figure 4.**
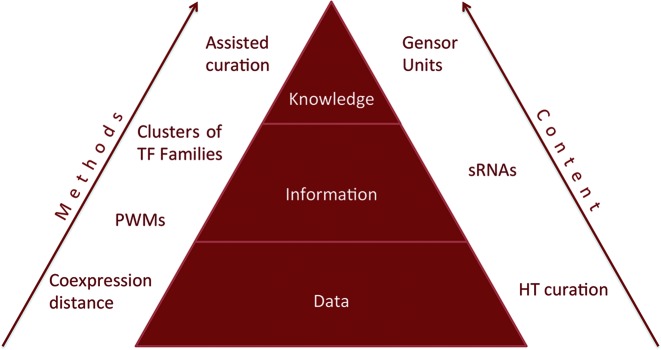
Schema of types of methods and content in RegulonDB.

RegulonDB is a complex evolving system. As mentioned in the ‘Introduction’ section, through the years we have gradually expanded both the content and level of detail of the biological data as well as the diverse types of experimental and bioinformatics sources of knowledge that nurture our understanding of gene regulation in *E.coli* K-12. A simplified diagram of our work is shown in Figure 4, with a triangle representing integration of data, information and knowledge; we do not mean absolute definitions of what is data or information, but simply provide relative concepts of the hierarchical nature of a highly granular knowledge. In the following discussion, we try to locate the major advances reported in this paper in this context.

For instance, the tsunami of HT-generated data is an effort that appears at the bottom, where decisions of what to represent where (datasets versus integrated sites in the database) reflect the tension of a classic paradigm of a reasonably well-organized genome, as opposed to one inundated by promoters and binding sites of unknown function.

Since the challenge of encoding this flux of knowledge is occurring faster than our human abilities and resources to keep our work up to date, we need to develop novel strategies. The assisted curation by means of NLP methodologies illustrates our efforts to implement strategies and test and improve them to accelerate our curation work. A direct benefit in years to come will be to have curated all specific contrasting conditions for each regulatory switch. Expansions to our work include the coexpression comparative metrics, enhanced collection of sRNAs regulation and clustering of PWMs and their grouping into TF evolutionary families. A landmark for this publication is the clear progress of the comprehensive collection of GENSOR units in an effort to enrich the top of the pyramid via overviews agglutinating large amounts of information that should make sense as a unit.

Briefly, we are guided by electronically editing in a structured way from the low granularity of details of mechanisms to the higher granularity regarding description and abstraction that offer broader perspectives to our understanding of the machinery and processes of *E. coli*′s way of life.

## SUPPLEMENTARY DATA

Supplementary Data are available at NAR Online.
